# A primary fish gill cell culture model to assess pharmaceutical uptake and efflux: Evidence for passive and facilitated transport

**DOI:** 10.1016/j.aquatox.2014.12.007

**Published:** 2015-02

**Authors:** Lucy C. Stott, Sabine Schnell, Christer Hogstrand, Stewart F. Owen, Nic R. Bury

**Affiliations:** a Division of Diabetes and Nutritional Sciences, King's College London, Franklin-Wilkins Building, 150 Stamford Street, London SE1 9NH, United Kingdom; b AstraZeneca, Alderley Park, Macclesfield, Cheshire SK10 4TF, United Kingdom

**Keywords:** 3Rs, reduce, refine, replace, A, apical, ABC, ATP-binding cassette, B, basal, BCF, bioconcentration factor, BTA, bi-directional transport assay, CETA, concentration equilibrated transport assay, Ci, Curie, Dpm, disintegrations per minute, *K*_ow_, octanol–water partitioning coefficient, MRP, multidrug resistance protein, OCT, organic cation transporter, OECD, Organisation for Economic Co-operation and Development, *P*_app_, apparent permeability coefficient, p*K*_a_, dissociation constant, QSARs, quantitative structure–activity relationships, REACH, Registration, Evaluation, Authorization & Restriction of Chemicals, SLC, solute carrier, TER, transepithelial resistance, TEP, transepithelial potential, TR, transport ratio, Animal alternatives, 3Rs, Pharmaceuticals, Bio-concentration, Fish, Rainbow trout

## Abstract

The gill is the principle site of xenobiotic transfer to and from the aqueous environment. To replace, refine or reduce (3Rs) the large numbers of fish used in *in vivo* uptake studies an effective *in vitro* screen is required that mimics the function of the teleost gill. This study uses a rainbow trout (*Oncorhynchus mykiss*) primary gill cell culture system grown on permeable inserts, which tolerates apical freshwater thus mimicking the intact organ, to assess the uptake and efflux of pharmaceuticals across the gill. Bidirectional transport studies in media of seven pharmaceuticals (propranolol, metoprolol, atenolol, formoterol, terbutaline, ranitidine and imipramine) showed they were transported transcellularly across the epithelium. However, studies conducted in water showed enhanced uptake of propranolol, ranitidine and imipramine. Concentration-equilibrated conditions without a concentration gradient suggested that a proportion of the uptake of propranolol and imipramine is *via* a carrier-mediated process. Further study using propranolol showed that its transport is pH-dependent and at very low environmentally relevant concentrations (ng L^−1^), transport deviated from linearity. At higher concentrations, passive uptake dominated. Known inhibitors of drug transport proteins; cimetidine, MK571, cyclosporine A and quinidine inhibited propranolol uptake, whilst amantadine and verapamil were without effect. Together this suggests the involvement of specific members of SLC and ABC drug transporter families in pharmaceutical transport.

## Highlights

• An *in vitro* model of the fish gill can be used to determine pharmaceutical transport across the gill.
• Propranolol uptake across this model is concentration and pH dependent and affected by inhibitors.
• A component of the uptake of some drugs is *via* a facilitated process.

## 1 Introduction

There are currently over 140,000 compounds that are being reassessed for their bioconcentrative properties as a part of the EU Registration, Evaluation, Authorization & Restriction of Chemicals (REACH) initiative (REACH, 2009). Conventionally, the main determinant used for assessing bioconcentration of a compound is the octanol–water partitioning coefficient (*K*_ow_), a measure of hydrophobicity that drives sorption and accumulation, and a main input parameter in quantitative structure–activity relationships (QSARs) (Hansch, 1969). However, this may not be fully applicable to pharmaceuticals, many of which are polar and ionizable (Hermens et al., 2013). In this case, the pH-corrected octanol–water partitioning coefficient, *D*_ow_, may be used, but this fails to take into account other major interactions such as hydrogen bonding and van der Waals forces, as well as uptake *via* carrier-mediated processes (Dobson and Kell, 2008, Sugano et al., 2010).

*In vivo* ecotoxicology testing produces bioconcentration factor (BCF) values that indicate the potential of a compound to bioconcentrate within an organism (OECD_305_, 2012). Fish are exposed to highly lipophilic compounds *via* the diet, whilst to others *via* the water; the principle being to use the uptake and depuration rates to calculate the propensity of a compound to bioconcentrate. Typically, each test can use up to 108 fish per compound and many thousands of fish are used for this test every year (Scholz et al., 2013). There is currently a desire to develop alternative methods to replace these standardized whole fish studies to recognize and classify environmental hazards (Creton et al., 2013, Wolf et al., 2007). This requires the identification and validation of appropriate *in vitro* systems that could replace such studies (Baron et al., 2012; Uchea et al., 2013). To find alternatives to the OECD_305_ water exposure it is necessary to identify a suitable fish gill model that mimics the intact organ because the gill, being constantly and continuously exposed to substances in water, is the principle site of xenobiotic uptake (Bury et al., 2014).

Fletcher et al. (2000) developed a double-seeding technique that enables primary gill cells to be cultured on permeable membrane inserts in a two-compartment model. This cultured epithelium comprises the different cell types (mitochondrial rich cells, respiratory cells and mucus cells) found in the gill and produces high transepithelial resistance (reviewed by Bury et al., 2014). Importantly, the system is able to tolerate apical freshwater and produces a negative transepithelial potential, further simulating the *in vivo* scenario. This is crucial when investigating the transport of ionizable compounds such as pharmaceuticals that may behave differently in culture medium and water. Furthermore, the gill cells from two fish can be used to create up to 72 individual gill epithelial inserts for assaying, thus potentially reducing numbers of fish in *in vivo* testing.

The present study thus aims to use the *in vitro* gill to investigate the uptake and efflux of seven pharmaceuticals representing a range of classes. We hypothesize that both passive transcellular and carrier-mediated transport of xenobiotics across the gill are likely principle drivers in determining the rate of uptake of waterborne compounds (Mckim and Erickson, 1991). Passive transcellular transport depends on the pH of the solution, acid–base constants (p*K*_a_) and the lipophilicity of the compound, whereas facilitated transport may be *via* members of the solute carrier (SLC) and ATP-binding casette (ABC) transporter families (Dobson and Kell, 2008). Therefore, to investigate carrier-mediated transport for some of these pharmaceuticals, concentration-equilibrated, pH-dependent, and concentration-dependent assays, as well as membrane channel inhibitor studies were conducted. In the context of this work, paracellular transport refers to the movement of compounds over membranes between cells and passive transport refers to concentration-dependent transcellular processes, whereas facilitated transport indicates concentration-independent carrier-mediated transport *via* membrane channel proteins. In addition, the uptake of propranolol across the *in vitro* gill model was compared to *in silico* and *in vivo* data (Owen et al., 2009), to demonstrate the use of this model as a predictive tool for pharmaceutical uptake.

## 2 Materials and methods

### 2.1 Animal husbandry

Gill cells for the use in primary cultures were obtained from juvenile diploid rainbow trout weighing 50–120 g purchased from a trout farm (Hampshire, UK). Fish were acclimatized in three 1000 L fiberglass aquaria at King's College, London, and maintained at 13–14 °C in recirculating aerated city of London tap water ([Na^+^] = 0.53 mM, [Ca^2+^] = 0.92 mM, [Mg^2+^] = 0.14 mM, [K^+^] = 0.066 mM and [NH_4_^+^] = 0.027 mM), which was passed through carbon, mechanical and biological filters. Photoperiod was maintained at a constant 14 h light/10 h dark cycle and fish were fed a daily 1% (w/w) ration of fish chow.

### 2.2 Gill cell culture

Sterile techniques were used throughout all cell culture procedures. Equipment, containers and solutions were autoclaved or sterile filtered (0.2 μm, Corning). The gill cell isolation procedure was based on methods previously documented (Fletcher et al., 2000) and the cell culture double-seeded insert (DSI) technique as described by Walker et al. (2008) and Wood et al. (2002). Briefly, primary gill cells are isolated, washed and resuspended in L-15 medium (Invitrogen) supplemented with FBS (5% (v/v)) (Sigma) and seeded onto a permeable polyethylene terephthalate (PET) membrane inserts with 0.4 μm pores with an area of 0.9 cm^2^ and maintained at 18 °C. This Transwell system (Corning) has an apical compartment above and a basal compartment below.

The development of an intact and electrically tight gill epithelium was monitored daily through ‘blank’-corrected measurements of transepithelial resistance (TER) using a custom-modified epithelial tissue voltohmeter (EVOMX; World Precision Instruments) fitted with chopstick electrodes (STX-2). The same device was used to measure transepithelial potential (TEP) before and after freshwater application. DSI epithelia that reached a TER of ≥5 kΩ cm^2^ were considered developed and electrically ‘tight’ for experimental procedures. In this instance, DSI preparations were washed twice with PBS (to remove any media supplemented with FBS) and exposed to radiolabeled pharmaceuticals apically in either L-15 medium (without FBS) or freshwater (2.0 mM CaCl_2_, 0.5 mM MgSO_4_, 0.8 mM NaHCO_3_, 77.1 μM KCl at pH 7.7), or basally, always in L-15 medium. L-15 medium has an osmolarity of 300–320 mOsm kg^−1^ (Invitrogen) and that of freshwater is around 15 mOsm kg^−1^. All experiments and exposures are based on individual inserts (*n*) derived from at least one biological replicate. Due to the seeding procedure over two days, one biological replicate is derived from two fish.

### 2.3 Membrane permeability

Paracellular permeability was measured using the paracellular marker ^14^C-mannitol (20 Ci mmol^−1^, Amersham Biosciences, CAS no. 88404-24-4). Thirty-seven DSI epithelia with TER values ranging from 0 to 14 kΩ cm^2^ were exposed to 0.013 μCi (2.2 × 10^5^ dpm) ^14^C-mannitol in 1.5 mL sterile freshwater in the apical compartment, with 2.0 mL L-15 medium in the basal (Hubatsch et al., 2007). From this, a TER value at which paracellular transport is at its most minimal can be deduced as a threshold for when epithelia are ready for transport assays (≥5 kΩ cm^2^). Aliquots of 100 μL were taken from the apical and basal compartments at time 0 and 24 h, and placed in 2 mL liquid scintillation fluid (Ecolume) and radioactivity measured by beta counting (Tri Carb 460CD liquid scintillation system; Packard). Mannitol flux after 24 h was calculated using Eq. (1):

(1) $$\text{Permeability}\;({\text{cm s}}^{-1})=\frac{{\left[\Delta M\right]}_{\text{BL}}\times \text{volume}}{M_{\text{AP}}\times \text{time}\times 3600\times \text{area}}$$

where [Δ*M*]_BL_ is the change in radioactivity in the basal compartment, *M*_AP_ is the radioactivity at the start, time is 24 h and area is 0.9 cm^2^ (Fletcher et al., 2000).

### 2.4 Radiolabeled pharmaceuticals

All drugs used in transport assays were at a concentration of 1 μg L^−1^ to represent the levels detected in the environment whilst remaining within detectable limits (Table 1). These were purchased radiolabeled and re-suspended in ethanol or methanol with a final solvent concentration in assay conditions of <0.0003%, and chosen to demonstrate a range of different classes (β_1_-, β_2_- and non-specific β-receptor agonists, a H_2_-receptor agonist and a tricyclic anti-depressant) with mid-range log *K*_ow_ values (see Table 1). This method of using labeled compounds allows for the recovery of label during cell-free conditions to calculate the amount that sticks to plastic ware. Furthermore, the label may be detected as either the parent compound or biotransformed products. ^3^H-propranolol hydrochloride (29.0 Ci mmol^−1^, CAS no. 152588-63-93) was obtained from Amersham Biosciences. ^3^H-metoprolol (29.7 Ci mmol^−1^), ^3^H-formoterol (18.5 Ci mmol^−1^) and ^3^H-terbutaline (29.0 Ci mmol^−1^) were obtained from Vitrax. ^3^H-Atenolol (7.3 Ci mmol^−1^) and ^3^H-ranitidine (2.5 Ci mmol^−1^) were obtained from Moravek Biochemicals, and ^3^H-imipramine hydrochloride (48.5 Ci mmol^−1^, CAS no. 113-52-0) from Perkin-Elmer.

**Table 1:** Properties of selected pharmaceuticals and the levels at which they are found in the environment.

Pharmaceutical	Use	MW	p*K*_a_	log *K*_ow_^1^	log *K*_ow_^2^	Environmental levels (ng L^−1^)
Propranolol	Non-selective β antagonist	259.340	9.4	2.54	1.12*	33^3^
Metoprolol	β1 receptor antagonist	267.364	9.6	1.76	–0.90#	410^3^
Atenolol	β1 receptor antagonist	266.336	9.6	0.67	0.0015*	940^3^
Formoterol	Long-acting β2 agonist	344.405	7.9^a^/9.2^b^	1.93	0.41*	n/a
Terbutaline	β2-Adrenergic receptor agonist	225.284	8.86^a^/9.76^b^	1.25	1.29	7^4^
Ranitidine	H2-receptor antagonist	314.4	8.08	1.47		120^3^
Imipramine	Tricyclic antidepressant	280.407	9.4	4.39		0.14^5^

1 Parameter Client (Tetko et al., 2005).

2 Environmental Risk Assessment data, experimentally measured values (AstraZeneca, 2014).

3 Kostich et al. (2014).

4 Breitholtz et al. (2012).

5 Giebułtowicz and Nałęcz-Jawecki (2014).

a Acidic.

b Basic

* pH 7.4.

# pH 7.

### 2.5 Bidirectional transport assays and apparent permeability coefficients

Bidirectional transport assays (BTA) assess both passive and facilitated transport in a bidirectional manner, from apical to basal (uptake) or *vice versa* (efflux). In these, concentration gradient conditions exist, whereby total transport is a sum of both passive transcellular and carrier-mediated processes. DSI epithelia with a TER > 5 kΩ cm^2^ with low paracellular transport rates were exposed to test compounds in either symmetrical or asymmetrical conditions. Symmetrical contained 1.5 and 2.0 mL L-15 (without FBS) in apical and basal compartments respectively, whilst asymmetrical required the application of 1.5 mL freshwater in the apical compartment and 2.0 mL L-15 in the basal. The test compound was added to either the apical side (uptake; A:B) or basal compartment (efflux; B:A) at a concentration of 1 μg L^−1^. Each experimental condition used 3–5 epithelial inserts from 1 to 2 biological replicates. For all experiments the water or media from the apical or basal compartment was mixed before taking 100 μL samples at 0, 6, 24, 30 and 48 h. Each 100 μL aliquot sample was placed into scintillation vials with scintillation fluid and beta-counted, and the drug concentration was calculated from the specific radioactivities. For uptake and efflux BTA, apparent permeability coefficients (*P*_app_) at 6 h were calculated using Eq. (2):

(2) $$P_{\text{app}}({\text{cm s}}^{-1})=\frac{\left(\text{d}Q/\text{d}t\times 1/(A\times C_{0})\right)}{3600}$$

where d*Q*/d*t* is the flux rate of the drug (pmol L^−1^ h^−1^), *A* is the surface area of the monolayer (0.9 cm^2^) and *C*_0_ is the initial concentration of the drug in the donor compartment (fM) (Petri et al., 2004). Transport ratios (TR) for both uptake and efflux were calculated using Eqs. (3), (4) (Schwab et al., 2003).

(3) $$\text{Uptake}\;\text{TR}=\frac{P_{\text{app}\;\text{A}:\text{B}}}{P_{\text{app}\;\text{B}:\text{A}}}$$

(4) $$\text{Efflux}\;\text{TR}=\frac{P_{\text{app}\;\text{B}:\text{A}}}{P_{\text{app}\;\text{A}:\text{B}}}$$

An uptake or efflux TR ≥ 1.5 is considered an indicator of active transport (Schwab et al., 2003, Luna-Tortós et al., 2008). For time-dependent BTA, the results were also expressed as the percentage of the initial drug concentration of the donor compartment for uptake (A:B) and efflux (B:A) over 48 h.

### 2.6 Concentration equilibrium transport assays

Concentration equilibrium transport assays (CETA) examine transport by adding equivalent concentrations on either side of the gill epithelium and assessing the movement of compounds over time to evaluate carrier-mediated uptake or efflux regardless of passive transport processes, as used in blood–brain barrier transport assays (Luna-Tortós et al., 2008). The same experimental procedures for BTA were used (in symmetrical and asymmetrical conditions), but with both apical and basal compartments containing test drugs at the same concentration of 1 μg L^−1^. The results are expressed as a percentage of the initial concentration in each compartment (apical or basal) over time.

### 2.7 The pH-dependent transport of propranolol

DSI epithelia were exposed in apical freshwater adjusted to pH 6 (by addition of HCl), pH 8 or pH 9.5 (by addition of NaOH; Corning pH meter 140). Radiolabeled propranolol was added at a concentration of 1 μg L^−1^ to either the apical or basal compartments (BTA) to investigate uptake and efflux. At 6 h a 100 μL sample was collected from the apical and basal compartments and radioactivity analyzed as above, and apparent permeability coefficients (*P*_app_) calculated using Eq. (2).

### 2.8 The concentration-dependent uptake of propranolol

DSI epithelia (*n* = 54) were exposed to 17 concentrations of propranolol ranging from 0.014 to 10,000 μg L^−1^. Concentrations above 0.1 μg L^−1^ were made using propranolol hydrochloride (Sigma, CAS no. 318-98-9) and radiolabeled propranolol as a marker. Experiments were conducted in asymmetrical conditions with propranolol added to the water in the apical compartment, thus mimicking the *in vivo* scenario (Owen et al., 2009). Radioactivity was analyzed as previously described in Section 2.3.

### 2.9 The inhibition of the uptake of propranolol

Cells were pre-incubated with inhibitor at a concentration 100 times higher (400 nM) than that of propranolol to competitively inhibit the membrane channel. Amantadine (Sigma, CAS no. 768-94-5), cimetidine (ICN, CAS no. 51481-61-9), cyclosporine A (Fluka, CAS no. 59865-13-3), MK571 (Tocris Biosciences, CAS no. 115104-28-4), quinidine (Sigma, CAS no. 56-54-2) or verapamil hydrochloride (Fluka, CAS no. 152-11-4) were dissolved in DMSO (0.1% in final solution) and added to the apical (in 0.75 mL freshwater) or basal (in 1.0 mL L-15 medium). Controls and compartments without inhibitor contained 0.1% DMSO. After 1 h, volumes were replaced with 1.5 mL freshwater containing 1 μg L^−1^ (4 nM) propranolol apically and 2.0 mL L-15 basally, whilst keeping the final concentration of inhibitor at 400 nM throughout. The same sampling procedure as for BTA at time 0, 6 (not for cyclosporine A), 24, 36 and 48 h proceeded and the uptake *P*_app_ of propranolol was calculated using Eq. (2). The *P*_app_ in all inhibitor-free controls were expressed as a percentage of the mean (100%) and the change in uptake *P*_app_ (inhibition) in the presence of inhibitors was expressed as a percentage of this mean control.

### 2.10 Analysis of data and statistics

For bidirectional *P*_app_ comparisons between the uptake and efflux, an independent samples *t*-test with equal variances assumed was used and statistical significance was accepted when *P* < 0.05 (SPSS software, SPSS Inc.). The same statistical analysis was used to test for differences between asymmetrical and symmetrical uptake or efflux *P*_app_ for each drug to assess the effect of apical freshwater application. For the time-dependent BTA significant differences between the uptake and efflux percentage of the donor compartment were tested for by one-way analysis of variance (ANOVA) on log-transformed data (SPSS software, SPSS Inc.). For the time-dependent CETA the statistical significance of differences between each percentage increase or decrease in the apical or basal compartments were also tested for by one-way ANOVA (after log transformation). Differences between the uptake of propranolol at different pHs, and similarly the efflux, were tested for by ANOVA and statistical significance was accepted when *P* < 0.05 (SPSS software, SPSS Inc.). The propranolol flux for each concentration in the concentration–response evaluation was calculated at 6 h and analyzed by ordinary least squares linear regression to describe the best fit, and further analysis of low concentrations was done by cubic polynomial regression to best describe the relationship (SPSS software, SPSS Inc.). Statistical differences between inhibitor (applied either apically or basally) and the inhibitor-free control were tested for by one-way ANOVA (after log transformation) and the statistical significance was set to *P* < 0.05.

### 2.11 Comparison to predicted and actual plasma concentrations

*In vitro* propranolol ‘internal’ concentrations after uptake (A:B) from the concentration–response study were compared to predicted *in silico* and actual *in vivo* plasma concentrations of propranolol in *Oncorhynchus mykiss*. The predicted partition of propranolol between blood and water can be determined by [plasma] = 0.87 [water] as described by Owen et al. (2009). This was calculated using the mammalian fish leverage model, whereby the predicted plasma concentration can be described by multiplying the environmental concentration by the blood to water partitioning coefficient (Huggett et al., 2004) using the Fitzsimmons model for the partitioning of compounds between blood and water (Fitzsimmons et al., 2001). Actual plasma concentrations of propranolol in *O. mykiss* were obtained from Owen et al. (2009). *In vitro* propranolol concentrations at 6 h (*n* = 3–6 from 3 biological replicates) were tested for correlation to *in silico* and *in vivo* plasma concentrations by ordinary least squares linear regression (SPSS software, SPSS Inc.)

## 3 Results

### 3.1 Epithelium membrane characteristics

**Fig. 1:**
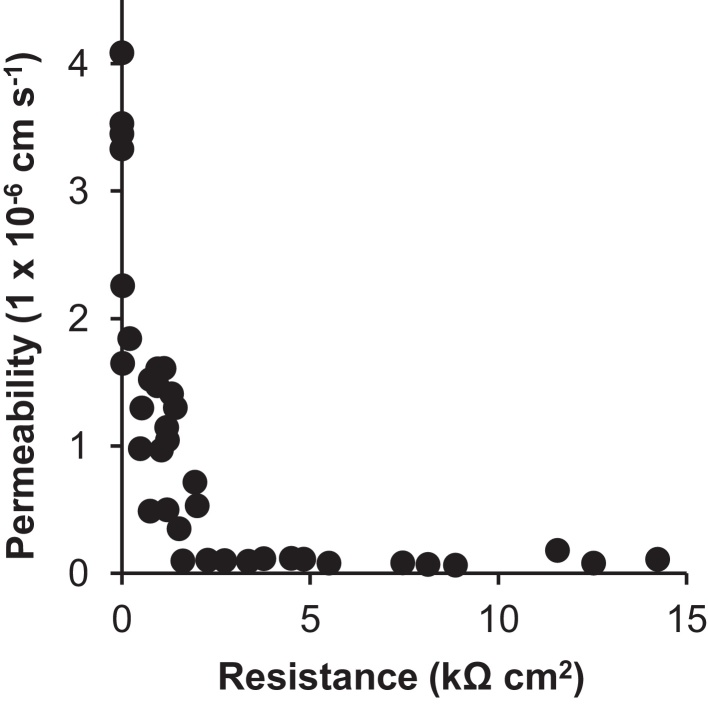
The relationship between ^14^C-mannitol permeability (after 24 h) and transepithelial electrical resistance (TER) in 37 cultured rainbow trout gill epithelia derived from four biological replicates. ^14^C-mannitol was applied to the apical compartment in freshwater, with L-15 medium in the basal compartment. Each data point represents one DSI epithelium (*n* = 37) from four biological replicates.

DSI epithelial development was monitored through daily measurements of TER in symmetrical conditions, which after 8 days reached 18.1 ± 1.3 kΩ cm^2^ (*n* = 24). After apical freshwater application TEP became increasingly negative from −0.9 ± 0.15 mV to −12.9 ± 2.9 mV (*n* = 8). The permeability of the paracellular marker ^14^C-mannitol produces a measure of the rate of paracellular transport for compounds with similar molecular weights *via* this route. Mannitol flux in cell free inserts with a TER of 0 Ω cm^2^ (blanked) was 3.6 ± 0.2 × 10^−6^ cm s^−1^ (*n* = 4, Fig. 1). DSI gill epithelia with TER of 200–1000 Ω cm^2^ exhibited a mannitol permeability of 1.1 ± 1.1 × 10^−6^ cm s^−1^, whilst those >2 kΩ cm^2^ showed the lowest mannitol permeability of 0.1 ± 0.01 × 10^−6^ cm s^−1^, with little variation between epithelia (Fig. 1).

### 3.2 Apparent permeability coefficients and transport ratios

**Fig. 2:**
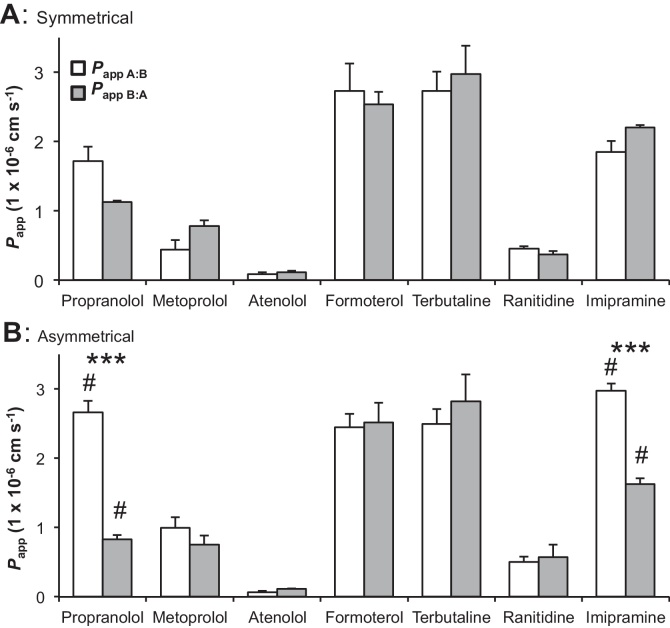
The apparent permeability coefficients (*P*_app_) for uptake (*P*_app__A:B_, white bars) and efflux (*P*_app__B:A_, gray bars) of seven pharmaceuticals (1 μg L^−1^) across the DSI gill cell epithelium at 6 h in symmetrical conditions (A) and asymmetrical conditions (B). Significant differences between the *P*_app__A:B_ and *P*_app__B:A_ for a drug within a condition (symmetrical or asymmetrical) are indicated by asterisk (independent samples *t*-test; ***P < 0.001). Significant differences between symmetrical and asymmetrical *P*_app__A:B_ or *P*_app__B:A_ for each drug are indicated by hash tag in (B) (independent samples *t*-test; ^#^*P* < 0.05). All experiments were performed in triplicate or more (*n* = 3–5) from at least one biological replicate and values are shown as means ± SEM.

In symmetrical conditions with L-15 medium on both sides of the gill cell epithelium no significant differences between *P*_app__A:B_ and *P*_app__B:A_ for all 7 pharmaceuticals was observed (Fig. 2A), yet all permeabilities were higher than that of the paracellular marker mannitol (except for atenolol; *P*_app__A:B_: 0.1 ± 0.03 × 10^−6^ cm s^−1^ and *P*_app__B:A_: 0.1 ± 0.02 × 10^−6^ cm s^−1^ compared to mannitol; *P*_app__A:B_: 0.1 ± 0.01 × 10^−6^ cm s^−1^). In asymmetrical conditions with freshwater at the apical surface of the gill epithelium, *P*_app__A:B_ was significantly higher than *P*_app__B:A_ (*P* < 0.001) for propranolol and imipramine, whilst no significant differences existed between uptake and efflux *P*_app_ of the remaining five drugs metoprolol, atenolol, formoterol, terbutaline and ranitidine (Fig. 2B). For propranolol, the *P*_app__A:B_ increased significantly from 1.7 ± 0.2 × 10^−6^ cm s^−1^ in symmetrical to 2.7 ± 0.2 × 10^−6^ cm s^−1^ in asymmetrical conditions (*P* < 0.05) and *P*_app__B:A_ decreased significantly from 1.1 ± 0.02 to 0.8 ± 0.1 × 10^−6^ cm s^−1^ (*P* < 0.05) (Fig. 2A and B). Similarly for imipramine a significant increase in *P*_app__A:B_ from symmetrical (1.9 ± 0.2 × 10^−6^ cm s^−1^) to asymmetrical (3.0 ± 0.1 × 10^−6^ cm s^−1^; *P* < 0.05) and a significant decrease in *P*_app__B:A_ (2.2 ± 0.04 to 1.6 ± 0.1 × 10^−6^ cm s^−1^; *P* < 0.05) (Fig. 2A and B) was observed.

Propranolol showed uptake TR values greater than 1.5 in both symmetrical and asymmetrical conditions, whilst for imipramine; this was only observed in asymmetrical conditions. Both metoprolol and atenolol had efflux TR values greater than 1.5 in symmetrical and asymmetrical conditions, respectively (Table 2).

**Table 2:** The uptake and efflux transport ratios (TR) of seven drugs in symmetrical and asymmetrical conditions.

	Uptake TR		Efflux TR	
	Symmetrical	Asymmetrical	Symmetrical	Asymmetrical
Propranolol	1.52*	3.21*	0.66	0.31
Metoprolol	0.56	1.32	1.78*	0.76
Atenolol	0.74	0.60	1.34	1.67*
Formoterol	1.08	0.97	0.93	1.03
Terbutaline	0.92	0.89	1.09	1.13
Ranitidine	1.21	0.88	0.83	1.14
Imipramine	0.84	1.83*	1.19	0.55

* represents TR ≥ 1.5.

### 3.3 Bidirectional transport assays

In symmetrical conditions with L-15 medium on both sides of the epithelium, ranitidine and imipramine exhibited greater efflux transport from the basal to the apical compartments at 24 h (*P* < 0.05) and at 30 and 48 h (*P* < 0.05) respectively. In asymmetrical conditions involving the application of freshwater at the apical surface, more propranolol, ranitidine and imipramine were taken up across the gill cell surface than effluxed after 48 h. This was significantly more so at 6 h (*P* < 0.001) and 24, 30 and 48 h (*P* < 0.01) for propranolol, 30 h (*P* < 0.01) for ranitidine and at all sampling points (6, 24, 30 and 48 h) for imipramine (*P* < 0.001). Metoprolol, formoterol and terbutaline showed slightly greater uptake than efflux after 48 h, and this was significantly more so for formoterol at 48 h (*P* < 0.05).

### 3.4 Concentration-equilibrated transport assays

**Fig. 3:**
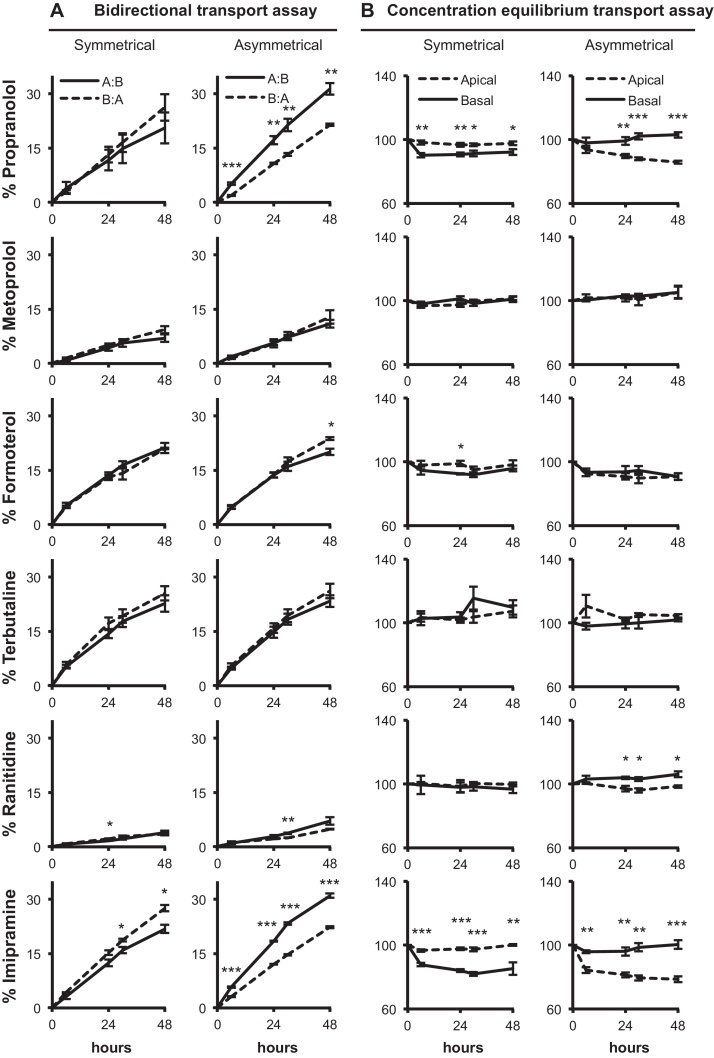
The transport assays of six pharmaceuticals: propranolol, metoprolol, formoterol, terbutaline, ranitidine and imipramine, in the DSI rainbow trout primary gill cell system over 48 h under (A) bidirectional transport assays (BTA) and (B) concentration equilibrium transport assays (CETA) conditions. For BTA (A) the drug is applied at a concentration of 1 μg L^−1^ to either the apical (–––) for apical to basal transport; uptake, A:B or basal compartment (– – –) for basal to apical transport; efflux, B:A in either symmetrical (L-15 medium in both compartments) or asymmetrical (freshwater in the apical compartment and L-15 medium in the basal) conditions. For this assay, data are shown as a percentage of the initial drug concentration in the donor chamber *versus* time. For CETA (B), the drug is added to both the apical and basal compartments at the same concentration (1 μg L^−1^) in both symmetrical and asymmetrical conditions. Data are shown as a percentage of the initial concentration in either the apical or basal *versus* time. For both assays, significant differences between the two compartments are indicated by asterisk (one-way ANOVA; *P < 0.05; **P < 0.01; ***P < 0.001). All experiments were performed in triplicate or more (*n* = 3–6) from at least one biological replicate and values are shown as means ± SEM.

In symmetrical conditions with L-15 media and test drug on both sides of the epithelium no significant differences between percentage of the initial drug concentrations in the apical and basal compartments were observed for metoprolol, terbutaline and ranitidine at any time point (Fig. 3B). The percentage of the initial concentration of formoterol was more in the apical than basal but only significantly so at 24 h (*P* < 0.05). The percentage of the initial concentration of propranolol in the apical compartment was significantly more than the basal at all sampling points after 0 h (6 and 24 h *P* < 0.01 and 30 and 48 h *P* < 0.05). This was also seen for imipramine, which showed the same increased basal to apical facilitated transport (*P* < 0.001 at 6, 24 and 30 h and *P* < 0.01 at 48 h). However, in asymmetrical conditions the situation was reversed whereby increased apical to basal transport, indicative of facilitated uptake, resulted in significantly more propranolol and imipramine in the basal compartments (*P* < 0.01 at 24 h and *P* < 0.001 to 30 and 48 h for propranolol and *P* < 0.01 at 6, 24 and 30 h and *P* < 0.001 at 48 h for imipramine). The same was true for ranitidine in asymmetrical conditions but to a lesser degree (*P* < 0.05 at 24, 30 and 48 h). Metoprolol, formoterol and terbutaline showed no signs of facilitated transport across the epithelium as no significant differences were observed (Fig. 3B).

### 3.5 The pH-dependent transport of propranolol

**Fig. 4:**
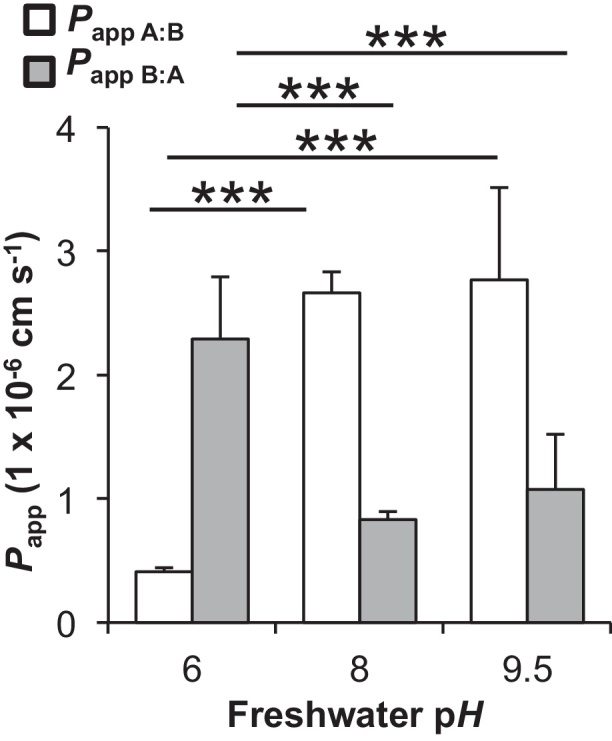
The pH-dependent uptake of propranolol. Shown are the apparent permeability coefficients (*P*_app_) for uptake (*P*_app__A:B_, white bars) and efflux (*P*_app__B:A_, gray bars) of propranolol (1 μg L^−1^) across the DSI gill cell epithelium at 6 h in asymmetrical conditions at three different pHs. Significant differences between uptake *P*_app_ or efflux *P*_app_ are indicated by asterisk (one-way ANOVA; ***P < 0.001). All experiments were performed in triplicate or more (*n* = 3–5) from two biological replicates and values are shown as means ± SEM.

A decrease in pH from 8 to 6 resulted in a significant reduction in the uptake permeability (*P*_app__A:B_) of propranolol from 2.7 ± 0.2 × 10^−6^ to 0.4 ± 0.03 × 10^−6^ cm s^−1^ (*P* < 0.001) in asymmetrical conditions. The effect was opposite for efflux permeability (*P*_app__B:A_) with an increase from 0.8 ± 0.1 to 2.8 ± 0.1 × 10^−6^ cm s^−1^ (*P* < 0.001; Fig. 4). An increase in pH from 8 to 9.5 was without significant effect (Fig. 4).

### 3.6 The concentration-dependent uptake of propranolol

**Fig. 5:**
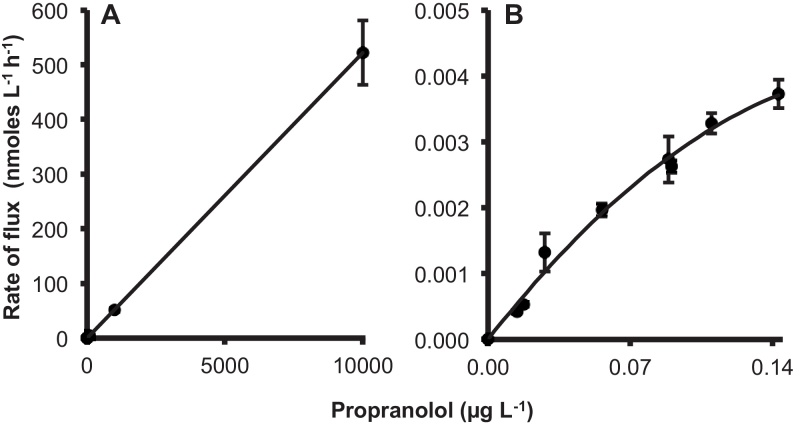
The concentration dependent uptake of propranolol (uptake; A:B) at 6 h after exposure to (A) 17 propranolol concentrations ranging from 0.014 to 10,000 μg L^−1^ with the line representing the fit of [rate] = 0.052 [concentration] (*n* = 54, *r*^2^ = 0.974) (note at lower concentrations that multiple data points are stacked) and (B) low propranolol concentrations (0.014–0.14 μg L^−1^) with the line representing the fit of [rate] = 0.057 [concentration] (*n* = 24, *r*^2^ = 0.927). The values represent mean ± SEM from five biological replicates.

A positive linear correlation between concentration (0.014–10,000 μg L^−1^) and flux of propranolol was observed with regression line representing the best fit of [rate] = 0.052 [concentration] (*n* = 54, *r*^2^ = 0.974, Fig. 5A). However, at lower propranolol concentrations (0.014–0.14 μg L^−1^) a 2nd order polynomial regression best described the concentration–response relationship (*n* = 24, *r*^2^ = 0.927, Fig. 5B) rather than linear (*n* = 24, *r*^2^ = 0.908) with line representing the best fit of [rate] = 0.057 [concentration].

### 3.7 The inhibition of the uptake of propranolol

**Fig. 6:**
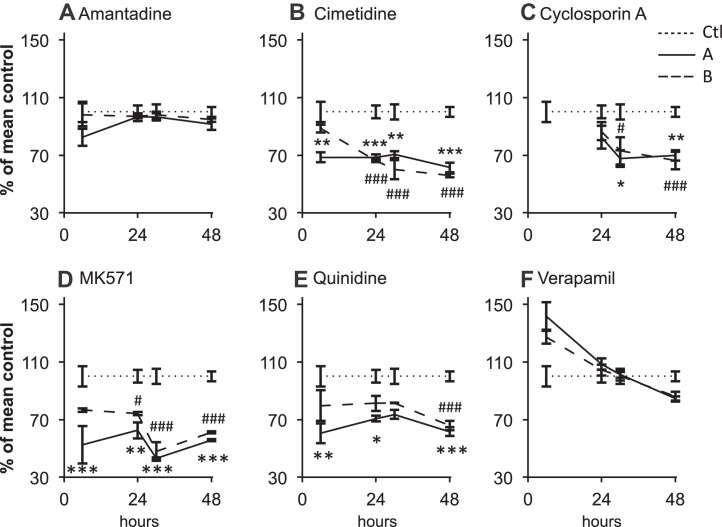
The inhibition of the uptake of 1 μg L^−1^ (4 nM) propranolol from apical (freshwater) to basal (L-15 medium) using 400 nM of the six inhibitors amantadine (A); cimetidine (B); cyclosporine A (C); MK571 (D); quinidine (E) and verapamil (F), applied either apically, AP (–––) or basally, BL (– – –). Data are shown as percentage of the mean control, CTL (⋯⋯) over time. Significant differences between the apically applied inhibitor and the control are indicated by asterisk (one-way ANOVA; *P < 0.05; **P < 0.01; ***P < 0.001). Significant differences between the basally applied inhibitor and the control are indicated by hash tag (^#^*P* < 0.05; ^##^*P* < 0.01; ^###^*P* < 0.001). All experiments were performed in triplicate or more (*n* = 3–6 from at least one biological replicate for inhibitor studies; *n* = 18 from six biological replicates for the inhibitor free controls) and values are shown as means ± SEM.

Amantadine and verapamil did not inhibit *P*_app__A:B_ of propranolol (Fig. 6A and F). Apical and basal cimetidine application significantly inhibited the transport of propranolol at all time points (apart from basal at time 6 h) by approximately 40–61.4% ± 3.4 (apical application) and 55.9% ± 1.3 (basal application) at 48 h (*P* < 0.001) (Fig. 6B). Similarly, cyclosporine A showed a significant inhibition of propranolol transport after 48 h to 69.9% (±3.7) of that in the control (*P* < 0.01) for apical application and to 66.3% (±6.1) (*P* < 0.001) for basal one (Fig. 6C). Application of the inhibitors MK571 and quinidine again caused a significant decrease in propranolol permeability over time by approximately 40% compared to the control at 48 h for both apical and basal applications (*P* < 0.001) (Fig. 6D and E).

### 3.8 Comparison to predicted and actual propranolol plasma concentrations

**Fig. 7:**
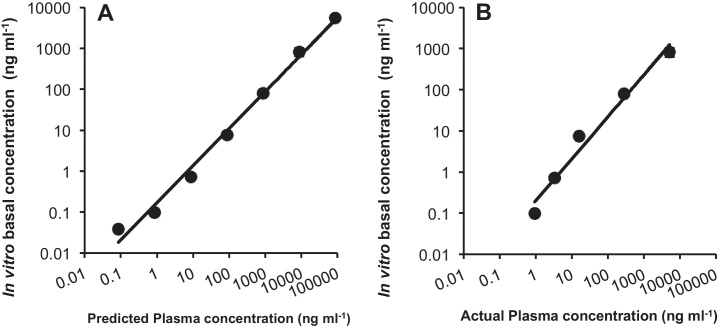
Propranolol concentrations in the basal compartment of the primary gill cell model after 6 h apical exposure to propranolol in asymmetrical conditions plotted against (A) predicted (see text) *Oncorhynchus mykiss* propranolol plasma concentrations (0.1–100,000 μg L^−1^) with regression line representing the fit of [*in vitro*] = 0.063 [predicted] (*n* = 21, *r^2^* = 0.995) or (B) actual *in vivo* plasma concentrations (1–10,000 μg L^−1^) after 40 days exposure (Owen et al., 2009) with the regression line representing the fit of [*in vitro*] = 0.155 [actual] (*n* = 21, *r^2^* = 0.966) (Table 3). Basal *in vitro* concentrations were performed in triplicate or more (*n* = 3–5) and represent mean ± SEM from three biological replicates.

Predicted plasma concentrations were calculated using [plasma] = 0.87 [water] and actual plasma concentrations from Owen et al. (2009) (Table 3). A linear correlation between predicted plasma concentration and *in vitro* basal concentrations was obtained and described by [*in vitro*] = 0.063 [predicted] (*n* = 21, *r*^*2*^ = 0.995, Fig. 7). Furthermore, a correlation between actual plasma concentrations (Owen et al., 2009) and *in vitro* basal concentrations was obtained and described by [*in vitro*] = 0.155 [actual] (*n* = 21, *r*^*2*^ = 0.966, Fig. 7).

**Table 3:** Propranolol concentration *in silico* (predicted), *in vivo* and *in vitro* (measured at various external propranolol concentrations).

Nominal propranolol concentration in water (μg L^−1^)	*In silico* (ng mL^−1^) (Huggett et al., 2004)	*In vivo* (ng mL^−1^) (Owen et al., 2009)	*In vitro* (ng mL^−1^)
0.1	0.087	n/a	0.04 (±0.002)
1.0	0.87	0.94* (n/a)	0.10 (±0.001)
10	8.7	3.3 (±0.4)	0.72 (±0.02)
100	87	16 (±7)	7.5 (±0.3)
1000	870	280 (±116)	79.5 (±7.3)
10,000	8700	5200 (±1333)	812 (±158)
100,000	87,000	n/a	5545 (±313)

* Pooled plasma sample.

## 4 Discussion

The development of suitable *in vitro* cellular models to replace, refine and reduce (3Rs) the numbers of fish used in ecotoxicological studies is an important requirement in current regulatory testing (Scholz et al., 2013). Pharmaceuticals in the environment are contaminants of emerging concern and their behavior in water affects uptake into biological systems (Boxall et al., 2012). Log *K*_ow_ is often used to determine the bioconcentration potential of a compound which may not be relevant to ionizable compounds such as pharmaceuticals (Hermens et al., 2013). The pH-corrected log *D*_ow_ is used to account for the fraction of ionizable and nonionisable species of a substance at a given pH, but this accounts for partitioning between two liquid phases and does not include partitioning across a biological membrane. The present study is the first to demonstrate how a primary rainbow trout gill cell culture system can be used to assess pharmaceutical uptake from water across a biological membrane. Therefore, this offers a potential alternative to replace whole organism pharmaceutical uptake studies at differing water chemistries, such as different pHs, the presence of dissolved organic matter or real water samples from the field.

Once a tight epithelium (>5 kΩ cm^2^) has formed, the gills present a barrier for paracellular transport that is relatively impermeable to the paracellular permeability marker ^14^C-mannitol (Fig. 1). All pharmaceuticals (except atenolol) were transported across the epithelium in both directions at a rate greater than that of ^14^C-mannitol, indicating that their transport is *via* a transcellular or carrier-mediated process, as all exhibit molecular weights greater than that of the marker (Hubatsch et al., 2007, Schwab et al., 2003).

The application of apical freshwater resulted in significantly different drug permeations for propranolol and imipramine where more is taken up across the epithelium from the water than effluxed from the basolateral compartment (Table 2; Fig. 2, Fig. 3). The uptake of ionizable chemicals such as pharmaceuticals depends on pH and the acid base constant (p*K*_a_) (Lahti et al., 2011). Both propranolol and imipramine are weak bases with p*K*_a_ values around 9.5. Using the Henderson–Hasselbalch equation, 99% of propranolol and imipramine exist in their protonated ionized form at pH 7.4 in symmetrical conditions, which then falls to only 98% in freshwater at pH 8. Here, at pH 8, more of the natural unionized forms are lipid-soluble, and thus may cross the membrane *via* passive transcellular routes explaining the enhanced uptake. However, the 1% change in speciation is unlikely to fully account for the difference in uptake observed (Fig. 2) and other factors are likely to play a role. For example, in symmetrical conditions TEP is positive (1.9 ± 0.2 mV) and becomes negative after apical freshwater application (−10.3 ± 0.2 mV, Fletcher et al., 2000; −12.9 ± 2.9 mV, data not shown), to a value similar to that observed *in vivo* (Potts, 1984). The change in membrane potential to basolateral negative generates an electrical gradient that aids cation partitioning across the membrane (Fletcher et al., 2000). Alternatively, the TR values greater than 1.5 in asymmetrical conditions for propranolol and imipramine in BTA conditions (Table 2) indicate that a proportion of the transport is *via* an carrier-mediated process (Schwab et al., 2003). This observation was substantiated by the concentration equilibrium transport assay (CETA) that eliminates concentration-dependent passive transport across the gill epithelium allowing the observation of carrier-mediated processes that drive xenobiotic transport. In CETA the difference in partitioning of radioactivity between the two compartments is not due to disproportional loss of compound adhering to the plastic ware of the apical and basolateral compartments, since in cell free blank insert experiments an equal percentage of the initial concentration added was absorbed to each compartment and an 80% recovery was obtained after 48 h (data not shown). Thus, it is possible to conclude that for propranolol and imipramine, facilitated transport makes up approximately 10% of the total transport, and around 5% for ranitidine efflux (Fig. 3B).

Uptake rates are important for predicting potential internal concentrations and are used to predict effects based on the “read across” hypothesis and Mode of Action (Rand-Weaver et al., 2013). Much of the *in vivo* uptake work used for these predictions expose fish to very high water concentrations and extrapolates back to these lower environmentally relevant values. Propranolol uptake is concentration-dependent over the whole range of concentrations but in the low, ng L^−1^ range, uptake deviates from linearity (0.014–0.14 μg L^−1^, Fig. 5). A similar result was obtained for the uptake at very low concentrations of iron across zebrafish gills and was attributable to proton-dependent metal transporters (Bury and Grosell, 2003). The facilitated transport of propranolol that occurs in these environmentally relevant concentrations (Kostich et al., 2014) is of interest because it suggests that the predictive models for uptake using data derived for higher concentrations may underestimate uptake.

The uptake of propranolol is pH dependent up to pH 8, after which further increases do not cause significant effects (Fig. 4). This again could be attributed to the difference in composition of ionized and unionized species at different pHs, with a caveat that a proportion of transport is likely *via* a facilitated process. However, it should be noted that the pH of the apical bulk compartment, and the microclimate at the boundary layer, were not measured after the 6 h duration of the experiment, and variations in such may account for changes in drug uptake. pH-dependent uptake of propranolol has been observed in other epithelia including retina (Kubo et al., 2013), Caco-2 cells (Wang et al., 2010) and kidney MDCK cells (Dudley et al., 2000). In contrast, efflux of propranolol is far greater at pH 6 than uptake (Fig. 4). This may suggest the export of propranolol or its metabolite is pH-dependent. Candidates for drug export are the ABC transporters; however these are not directly regulated by pH changes (Altenberg et al., 1993, Neuhoff et al., 2003). The internal pH of the cells is constant against external pH changes and thus intracellular speciation of the drug is unlikely to explain potential increase in efflux. It is likely that there are other propranolol parent and/or metabolite exporters present on the gill.

Propranolol uptake from water was inhibited by cimetidine, cyclosporine A, MK571 and quinidine. These are inhibitors of a number solute carrier and ABC transporters. A number of human SLCs implicated in drug transport (SLC15s, SLCOs, SLC22s and SLC47s [Dobson and Kell, 2008, Giacomini et al., 2010]) are homologous to SLCs found in teleost fish (Verri et al., 2012). Similarly, ATP-binding cassette (ABC) transporters involved in the cellular efflux of toxicants such as ABCBs, ABCCs and ABCG2 (Deeley et al., 2006) are considered highly conserved amongst vertebrates (Dean and Annilo, 2005) and have been documented in rainbow trout cells both *in vivo* and *in vitro* (Fischer et al., 2011, Lončar et al., 2010) but not fully characterized. Recent microarray studies using the primary gill cell culture system have indentified the presence of transcripts for genes encoding a number of SLCs, ABCs as well as biotransformation enzymes (Schnell, Bury, Kille and Hogstrand, unpublished data). But, the identification of active proteins requires further work.

The application of cimetidine and quinidine, which both interact with organic cation transporter OCT2 (SLC22A2) (Giacomini et al., 2010, Koepsell, 2013) significantly reduce propranolol uptake. Propranolol transport *via* OCT2 was observed in renal LLC-PK_1_ cells transiently transfected with hOCT2-V5, more specifically in the active uptake of its cationic form across the apical membrane (Dudley et al., 2000). Cimetidine is also used as a blocker of cisplatin transport by OCT2 in zebrafish lateral line hair cells (Thomas et al., 2013) and the presence of OCT2-like proteins have been suggested in other teleost fish gill epithelia (Verri et al., 2012). MK571 is an inhibitor of the multidrug resistance protein (MRP) efflux pumps (Deeley et al., 2006) and significantly inhibited propranolol uptake. The main MRP expressed in the gill is MRP3 (ABCC3) (Lončar et al., 2010). P-glycoprotein (Pgp; ABCB1) is implicated in propranolol transport in rabbit conjunctivial epithelial cells (Yang et al., 2000) and Caco-2 cells (Wang et al., 2010) and is present in rainbow trout tissues but at low levels in the gill (Lončar et al., 2010). Whether Pgp is involved in propranolol transport in the gill cell culture system is unclear. In addition to inhibiting OCT2, quinidine also acts as Pgp inhibitor (Giacomini et al., 2010) and cyclosporine A, another Pgp inhibitor, also blocked propranolol transport but this may be *via* MRP inhibition instead. Application of the Pgp inhibitor verapamil, however, did not affect propranolol transport (Fig. 6F). Taken together these observations would suggest that OCT2 and MRP3 may be candidates for propranolol transport across the teleost gill epithelium, but the location (apical or basolateral membrane) of these transporters, and others, needs further assessment.

Owen et al. (2009) showed that predicted plasma concentrations (Fitzsimmons et al., 2001) were good indicators of propranolol uptake over a range of high concentrations. Our data also correlates well with predicted (Fig. 7A) and measured plasma concentrations (Fig. 7B). However, *in vitro* propranolol concentrations in the basolateral compartment were an average 6% of the predicted and 16% of actual plasma concentrations, whilst actual plasma concentrations were only 59% of predicted (Owen et al., 2009). *In vivo* bioconcentration studies can involve an uptake phase of 60 days until a steady state is reached (OECD_305_; OECD, 2012). Owen et al. (2009) used a 40-day exposure period, and both procedures use a flow through system with a steady-state endpoint. Our *in vitro* assay took place over 6 h in a static system, which could account for reduced propranolol uptake. Furthermore, actual plasma concentrations may be lower than predicted as a proportion of the drug may bind to proteins or be metabolized by the gill (as suggested by Bartram et al., 2011), which prediction models fail to take into account. Nevertheless, the relationships between this *in vitro* system to predicted and actual propranolol plasma concentrations suggest its applicability as a suitable *in vitro* model to investigate the uptake of xenobiotics and when combined with elimination rates, may supplement *in vivo* bioconcentration fish studies. These model pharmaceuticals used do not bioaccumulate; however, they may be taken into a fish, and or excreted. This functional primary gill model facilitates our better understanding of these processes. Currently legislation requires assessment for compounds of log *K*_ow_ higher than 3. This is an arbitrary value and we suspect that this value may be conservative with little prospect of significant bioaccumulation for compounds lower than log *K*_ow_ 4. This model provides a rapid and ethically acceptable tool with which to perform a preliminary assessment of compounds of low log *K*_ow_, and as such ideal for large numbers of pharmaceuticals that typically have a lower propensity to bioaccumulate.

The study shows that as well as the passive uptake and efflux of neutral forms of pharmaceuticals, water enhances drug uptake of ionizable pharmaceuticals *via* both passive transcellular and carrier mediated processes. Hence, the ability of this system to tolerate freshwater is fundamental if we are to simulate *in vivo* drug uptake across the gill. In addition, the facilitated uptake of propranolol was more evident at low concentrations that are more environmentally relevant; suggesting that in certain situations uptake may be under predicted. Indeed there is significant variation (five-fold) recorded in plasma concentrations of individual fish exposed to pharmaceuticals *in vivo* (Owen et al., 2009), and it could be that individual difference in transporter expression may be a mechanistic explanation for this variance. The use of this system provides an opportunity to reduce the numbers of fish used in regulatory ecotoxicological testing as the primary gill cells cultured from two fish may provide up to 72 individual gill cell cultures, and this method bypasses *in vivo* drug exposures and uses much less test compound, thus offering refinement through improved animal welfare methods.

## Conflict of interest statement

The authors declare no financial conflict of interest.
